# INVESTIGATING THE RELATIONSHIP BETWEEN INTERNET USE AND PERCEIVED LONELINESS AMONG OLDER CHINESE

**DOI:** 10.1093/geroni/igz038.1538

**Published:** 2019-11-08

**Authors:** Shijie Song, Shijie Song, Yuxiang (Chris) Zhao, Qinghua Zhu

**Affiliations:** 1 Institute of Health Informatics, School of Information Management, Nanjing University, Nanjing, Jiangsu, China; 2 Nanjing University, Nanjing, Jiangsu, China (People’s Republic); 3 Nanjing University of Science and Technology, Nanjing, Jiangsu, China (People’s Republic)

## Abstract

There is a long debate on the impact of Internet use on individuals’ perceived loneliness. This study aimed to investigate the relationship between older Chinese people’s Internet use and their perceived loneliness. We employed cross-sectional data from the 2015 China Health and Retirement Longitudinal Study (N = 7,343; age range: 50-80 years old; Mean = 62.48; SD=8.00). Loneliness was measured by a 4-point Likert scale (Mean = 1.60, SD = 1.05) on the frequency of loneliness perception (higher score indicates higher loneliness). Internet use was measured by a dummy variable (Mean = 0.05, SD = 0.23). The results of multiple regression suggest that older Internet users reported significantly lower loneliness (B = -0.127, SD = 0.045, p = 0.005) compared with non-Internet users, suggesting a mitigating effect of Internet use on loneliness. Thus, the Internet might be implemented as an intervention to reduce loneliness among older adults in China.

